# Diagnostic Accuracy of Lung Ultrasound for Pneumonia in Adult Intensive Care Unit Patients: A Systematic Review and Meta-Analysis

**DOI:** 10.7759/cureus.115858

**Published:** 2026-09-06

**Authors:** Kelechi Meremikwu, Sean Lee, Brandon Tang, Melissa L Wilson, Keith Killu

**Affiliations:** 1 Department of Medicine, Division of Pulmonary, Critical Care and Sleep Medicine, University of Southern California, Los Angeles, USA; 2 Department of Medicine, Keck School of Medicine, University of Southern California, Los Angeles, USA; 3 Southern California-Clinical and Translational Science Institute, Keck School of Medicine, University of Southern California, Los Angeles, USA

**Keywords:** critically ill adult patients, intensive care unit, lung ultrasound, pneumonia, point-of-care ultrasound (pocus)

## Abstract

This systematic review and meta-analysis were conducted in an effort to evaluate the accuracy of using lung ultrasound (LUS) in the diagnosis of pneumonia in adult patients who are admitted to the intensive care unit (ICU) compared to the standard of care. The search involved obtaining data through the Medical Literature Analysis and Retrieval System Online (MEDLINE), PubMed, ClinicalTrials.gov, and Google Scholar and was conducted from inception until May 2026 to identify eligible studies. We included adult patients admitted to the ICU who were diagnosed with pneumonia on admission or developed pneumonia while in the ICU as hospital-acquired pneumonia (HAP)/ventilator-associated pneumonia (VAP). Those patients were evaluated by bedside LUS for the diagnosis of pneumonia, and their results were compared to the standard of care.

A total of 2,704 articles were identified. Six studies were included in this systematic review and meta-analysis, presenting a total of 518 patients enrolled and 275 patients diagnosed with pneumonia using the standard of care, which included chest X-ray (CXR), CT scan of the chest, laboratory and culture data, biomarkers, or clinical diagnosis by the provider. The mean age was 63.9 years. 71.2% of patients were male, and 28.8% were female. The estimated summary measures of sensitivity (SE) and specificity (SP) of LUS were calculated as 0.95 (95% CI: 0.88, 0.98, p<0.01) and 0.71 (95% CI: 0.60, 0.80, p<0.01), respectively. Heterogeneity was moderate for both SE (I2=32.68, 2=0.38) and SP (I2=13.17, 2=0.15). The LUS application in the ICU has a high SE and moderate SP in the diagnosis of pneumonia in adult patients compared to the standard of care. Larger cohorts and data collection with analysis are needed to further understand and optimize protocols for LUS in the ICU to better inform the management of patients with pneumonia.

## Introduction and background

Pneumonia is one of the most common infections with high morbidity and mortality, affecting all ages, in particular older adults worldwide, with an estimated one million deaths annually in patients above 70 years of age [1, 2]. Pneumonia in the ICU has a high burden, with a mortality rate of severe pneumonia estimated to be around 20%-50% [3]. Many patients with severe community-acquired pneumonia (CAP) are admitted to the ICU; others develop hospital-acquired pneumonia (HAP) or ventilator-associated pneumonia (VAP) [4] while in the ICU. Due to the high risk of morbidity and mortality, it is imperative to establish a prompt diagnosis and initiate the proper treatment. 

How the diagnosis of pneumonia is made across different parts of the world can vary and may depend on the regions’ resources and guidelines [5]. The standard in evaluation, assessment, and management of patients with suspected pneumonia in the ICU depends on many factors, including particular attention to the history, physical exam, obtaining laboratory studies, cultures, specific biomarkers, chest X-ray (CXR), and in many instances a CT scan of the chest [6]. Lately, the updated guidelines from the American Thoracic Society (ATS) address the diagnosis of patients with CAP and the possibility of using lung ultrasound (LUS) to aid in the diagnosis in centers where clinical expertise exists [7].

The application of LUS in the ICU has been increasing over the last two to three decades, with advancements in machine quality, operator training, and evolving guidelines. LUS availability as a bedside technology, combined with the reproducibility of its results, makes it a practical and valuable tool that aids clinicians in the diagnosis of pneumonia, especially when practicing in a busy ICU or in resource-limited settings where other modalities for diagnosis are not readily available [8].

Studies on the use of LUS in non-ICU patients and its accuracy compared to CXR, CT scan of chest, clinical and laboratory results for CAP have been conducted, as well as meta-analyses comparing LUS accuracy in the diagnosis of pneumonia in adult and pediatric patients [9-12], with consistent positive results indicating that LUS is accurate or superior to conventional methods. Observational studies examining the correlation between LUS and the standard of care in the diagnosis of pneumonia in the adult ICU population are limited. The adult ICU population presents different challenges in the diagnosis of pneumonia due to the severity of their illness, along with other comorbidities, and the technical challenges of performing ultrasound on supine patients in most instances, where many patients are on mechanical ventilation, under sedation, and with hemodynamic instability. One recent meta-analysis compared LUS to specifically CXR [11] in adult ICU patients but did not specifically compare LUS to all other diagnostic modalities for pneumonia in ICU adult patients.

Our aim in this systematic review and meta-analysis is to evaluate studies of specifically adult critically ill patients who are admitted to the ICU with pneumonia by using bedside LUS and compare its findings to the standard of care (including any combination of the following: history, physical exam, laboratory studies, cultures, biomarkers, CXR, and on many occasions a CT scan of the chest). This approach reflects real-world variability and applicability where different standards for the diagnosis of pneumonia exist. 

## Review

Materials and methods

This Systematic review and meta-analysis were registered in the Prospective Register of Systematic Reviews (PROSPERO 2026 CRD420261388874). The reporting of the present review and meta-analysis closely followed the Preferred Reporting Items for Systematic Reviews and Meta-Analyses (PRISMA) statement.

Population, Intervention, Comparator, and Outcome (PICO) Framework

The PICO framework was used for conducting the research and framing the research question; P: Adult patients in the ICU with a diagnosis of pneumonia; I: Performing LUS on adult patients with pneumonia in the ICU for diagnosis; C: Adults in the ICU with a diagnosis of pneumonia using the standard of care (including all or some of the following: history, physical exam, laboratory studies, cultures, specific biomarkers, CXR and on many occasions a CT scan of the chest). O: Accuracy of LUS in the diagnosis of pneumonia compared to the standard of care

Inclusion Criteria

Studies about adult patients admitted to the ICU who had a diagnosis of severe CAP, HAP, or VAP and where LUS was performed, and the results compared to the standard of care used for the diagnosis of pneumonia were included in the review.

Studies that included patients admitted to the ICU for severe pneumonia confirmed by one or more of the following: CXR, CT scan of the chest, laboratory and culture data, or clinical diagnosis by the provider. These modalities are considered the standard of care of practice in most ICU settings. Enrolled patients also underwent LUS, and the results were compared to the corresponding standard of care. Retrospective and prospective as well as cross-sectional studies were included if patients diagnosed with pneumonia using the standard methods underwent LUS examination to allow for direct comparison of results. 

Exclusion Criteria

Studies that included children or non-ICU adult patients and those that did not report a correlation between the standard of care and LUS in the diagnosis of pneumonia were excluded. 

Data Sources and Search Strategy

A comprehensive search of literature was conducted in Medical Literature Analysis and Retrieval System Online (MEDLINE), Excerpta Medica database (Embase), and Web of Science. All publications available in each database were included in the search. The search strategy was created by reviewing medical subject headings and MeSH terms (see below) relevant to the area of interest. Accordingly, key search terms were extracted from known, relevant, prominent studies. A combination of search terms was constructed, then a search was conducted to ensure we included a set of known specific articles. Key reviews in the area were screened for additional relevant references.

MeSH terms used for the search included the following: "Pneumonia; Ultrasonography; Intensive Care Units"; ((((((((("Pneumonia"[Mesh:NoExp]) OR "Bronchopneumonia"[Mesh]) OR "Healthcare-Associated Pneumonia"[Mesh]) OR "Pleuropneumonia"[Mesh]) OR "Pneumonia, Ventilator-Associated"[Mesh]) OR "Pneumonia, Aspiration"[Mesh]) OR "aspiration pneumonia"[tw] OR "Community-Acquired Pneumonia"[Mesh]) OR "Pneumonia, Bacterial"[Mesh]) OR "Pneumonia, Viral"[Mesh] OR "pneumonia*"[tw] OR "lobar pneumon*"[tw] OR "pneumonitis"[tw] OR "lung inflammat*"[tw] OR "inflammat*, lung"[tw] OR "pulmonary inflammat*"[tw] OR "inflammation, pulmonary"[tw] OR "hospital acquired pneumonia"[tw] OR "lung infiltrate"[tw] OR "organizing pneumonia"[tw] OR "bronchiolitis obliterans organizing pneumonia"[tw]) AND ("Ultrasonography"[Mesh:NoExp] OR "diagnostic ultraso*"[tw] OR "ultras*, diagnostic"[tw] OR "echography"[tw] OR "medical sonograph*"[tw] OR "sonography, medical"[tw] OR "ultraso* imaging"[tw] OR "imaging ultraso*"[tw] OR "ultrasonic tomography"[tw] OR "tomography, ultrasonic"[tw] OR "LUS"[tw] OR "lung ultraso*"[tw] OR "chest ultraso*"[tw] OR "thoracic ultraso*"[tw] OR "lung sonograph*"[tw] OR "chest sonograph*"[tw] OR "thoracic sonograph*"[tw])

Selection Process

Reviewers screened study titles and abstracts for eligibility. Three independent reviewers determined whether to include each study in the review, and, in the case of conflict, an independent reviewer adjudicated. The decision, as well as the reasons for inclusion or exclusion, are recorded on the PRISMA flow diagram. Four reviewers used the Quality Assessment of Diagnostic Accuracy Studies (QUADAS-2) [13] to rate the vulnerability of each included study to bias based on how patient selection was done, the index testing, whether the reference standard was followed, and flow and timing. When discrepancies occurred, a consensus among all reviewers was established. 

Harmonization and Extraction of Data

The principal investigators and all authors reviewed each study’s full text and extracted the data items of interest. In the event of a conflict, the article was adjudicated by all authors for final inclusion.

Statistical Analysis

We performed a random-effects meta-analysis and meta-regression to derive summary estimates of sensitivity (SE) and specificity (SP) using numbers of true positives, false positives, true negatives, and false negatives. We evaluated heterogeneity using I² and assessed small study effects, publication bias, and asymmetry using Deeks’ plot and Fagan’s test. To investigate heterogeneity, we performed meta-regression with each of the following covariates: study design, LUS protocol, age, LUS compared to CXR alone, LUS compared to CT scan alone, and whether the study included patients on mechanical ventilation (MV). All analyses were conducted using Stata 18 (Stata Corp., College Station, TX, USA), and p≤0.05 was considered statistically significant. 

Results

Study Selection and Search Results

**Figure 1:**
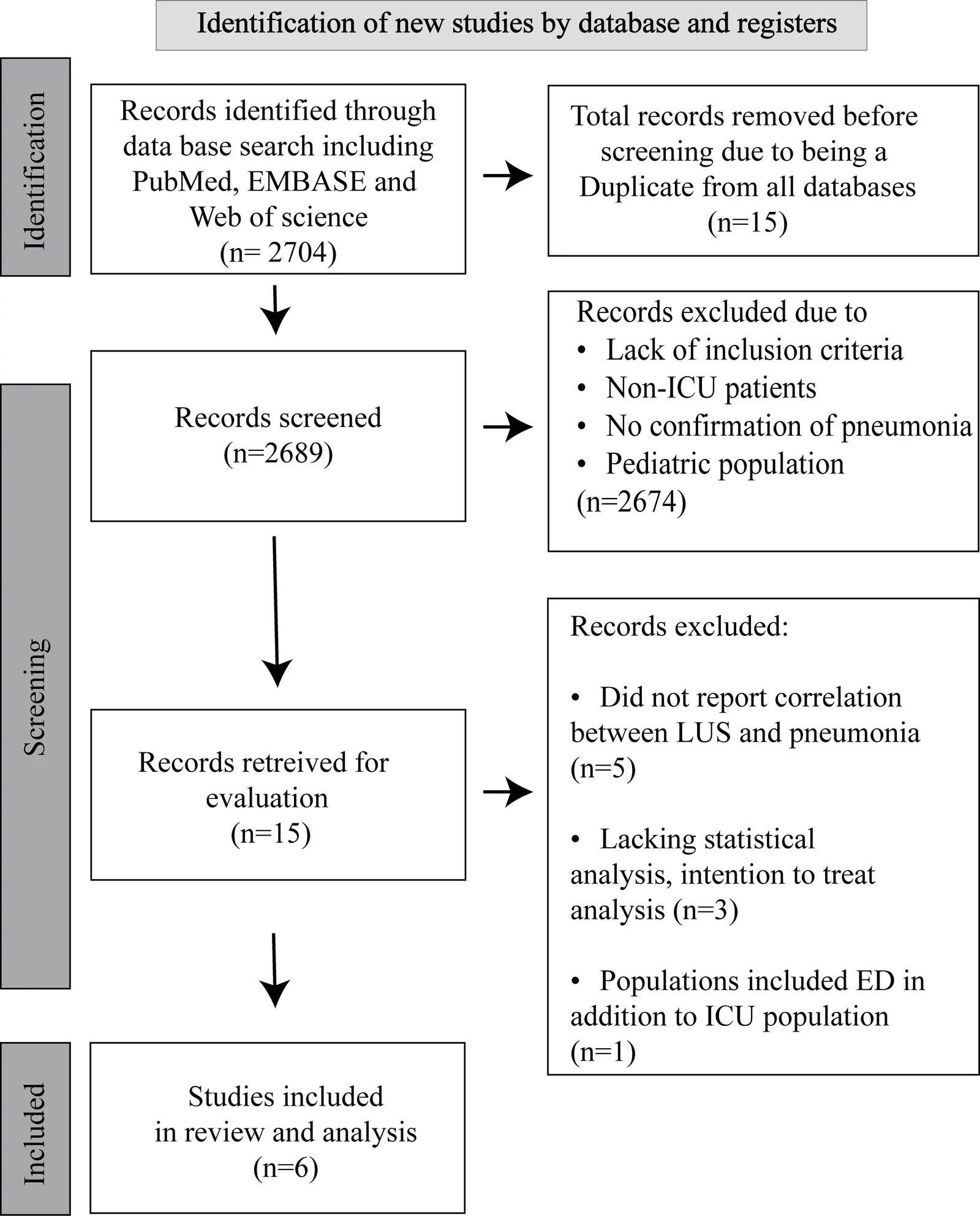
PRISMA flow diagram outlining the study selection process. This figure shows that the identification process, as well as screening and eligibility, were done based on the PRISMA guidelines for systematic reviews and meta-analyses. PRISMA: Preferred Reporting Items for Systematic Reviews and Meta-Analyses; ICU: intensive care unit; ED: emergency department; Embase: Excerpta Medica database; LUS: lung ultrasound

The search was conducted between November 2024 and November 2025, and 2,704 articles were identified. A total of 2,689 articles were excluded after the removal of duplicates and articles that didn’t meet the inclusion criteria. Fifteen studies were fully reviewed. Nine studies were excluded from the review, where five studies reported only on the progression and severity of the disease and not the initial diagnosis of pneumonia in the ICU [14-19]. Two studies were excluded due to lack of reporting on the correlation of LUS results and the standard of care and statistical analysis [19, 20]. One study was excluded due to lack of clarity about the number of patients who were in the ICU [21]. One study was excluded due to lack of the intention to treat in analysis [22]. Six studies were included in the review and meta-analysis (Figure 1) [23-28]. 

A total of 518 patients were enrolled across all six studies, and 275 patients were diagnosed with pneumonia using the standard of care. All studies included were in a single ICU, except one involving multiple ICUs. Study types were mixed, with one cross-sectional and five prospective cohorts. All studies used paired data on the same patient in the standard care and LUS. The average age was 63.9 years; 71.2% of patients were male, and 28.8% were female. No study included reports on the patients’ ethnicity. MV reporting varied: three studies included all patients on MV, one study included part of their cohort on MV, and two studies had no documentation regarding MV. There were no unified protocols for performing LUS. Two studies did not report on the protocol used; one study used 10 area zones on the chest, and three studies used 12 area zones for LUS exams. There were differences in reporting the diagnosis of pneumonia using the standard of care, where a combination of clinical data, including signs, symptoms, laboratory studies, CXR, and CT chest, was reported and used for the diagnosis. Different ultrasound machines and transducers were used (Table 1). Bias assessment using the QUADAS-2 tool was performed and is shown in Table 2.

**Table 1:** Characteristics of included studies and their patient populations LUS: lung ultrasound; VAP: ventilator-associated pneumonia; MV: mechanical ventilation; LOS: length of stay; CXR: chest X-ray; PCT: procalcitonin; CPIS: Clinical Pulmonary Infection Score

Author	Country	Study design	Total number of patients	Number of patients with pneumonia	Study participants	Patients on MV	Standard used for diagnosis of pneumonia	Primary outcome	Secondary outcome
Babu et al., 2025 [23]	India	Prospective observational	50	31	Average age= 56.9 years; Male=24, Female=7	50	Clinical exam, biological test	Diagnostic accuracy of LUS in identifying VAP	Days of MV, LOS in the ICU, hospital LOS and mortality
Bertlet et al., 2015 [24]	Switzerland	Prospective observational	57	12	Average age= 61.3 years; Male=34, Female=23	57	Clinical exam, biological test, CXR, Chest CT	Correlation between LUS and diagnosis of pneumonia	Not specified
Bitar et al., 2019 [25]	Kuwait	Prospective observational	92	73	Average age= 68.3 years; Male=31, Female=42	Not specified	Clinical exam, biological test, CXR, Chest CT	Correlation between LUS and diagnosis of pneumonia	Not specified
Dhawan et al., 2022 [26]	India	Cross-sectional	85	85	Average age= 55.9 years; Male=56, Female=29	36	Clinical exam, biological test, CXR, Chest CT	Diagnostic accuracy of LUS compared to CXR for diagnosis of pneumonia, and using CT as the gold standard	Characteristic LUS patterns in pneumonia and their frequencies
Mancusi et al., 2022 [27]	Italy	Prospective observational	110	26	Average age= 72; Male=21, Female=5	Not specified	Clinical exam, biological test, CXR, Chest CT	Diagnostic accuracy of LUS compared with CXR for detecting pneumonia	Diagnostic performance of LUS compared with CT scan. Effect of pleural effusion on diagnostic accuracy. Combined diagnostic performance of LUS plus C-reactive protein
Zhou et al., 2019 [28]	China	Prospective observational	124	48	Average age= 69; Male=30, Female=18	124	Chest CT, respiratory cultures, PCT	Diagnostic accuracy of LUS combined with PCT for diagnosis of VAP.	Accuracy of LUS alone, accuracy of PCT alone, accuracy of CPIS

**Table 2:** Quality and bias assessment following the Quality Assessment of Diagnostic Accuracy Studies (QUADAS-2) tool. Low: low risk; High: high risk; Unclear: unclear risk

Study	Risk of bias				Applicability Concern		
	Patient selection	Index test	Reference standard	Flow & timing	Patient selection	Index test	Reference standard
Babu et al., 2025 [23]	Low	Unclear	High	Low	Low	Unclear	High
Bertlet et al., 2015 [24]	Low	Low	Unclear	Low	Low	Low	Unclear
Bitar et al., 2019 [25]	Low	Unclear	Low	Low	Low	Unclear	Low
Dhawan et al., 2022 [26]	Low	Unclear	Low	Low	Low	Unclear	Low
Mancusi et al., 2022 [27]	Low	Low	Low	Low	Low	Low	Low
Zhou et al., 2019 [28]	Low	Low	Unclear	Unclear	Low	Low	Unclear

Summary of estimates of LUS accuracy

**Figure 2:**
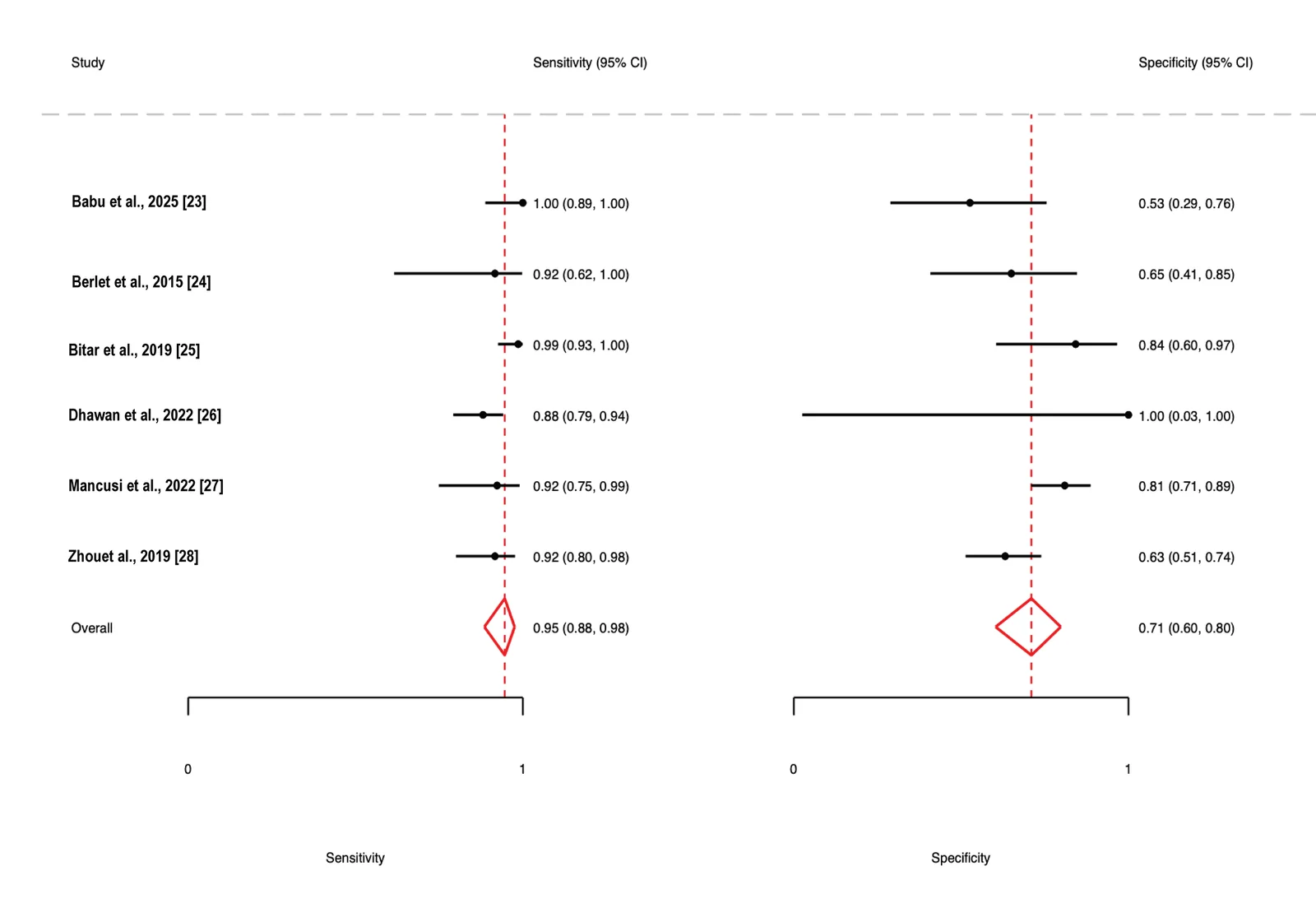
This forest plot provides the summary estimates for sensitivity and specificity, showing total and effects for each study included. CI: confidence interval

**Figure 3:**
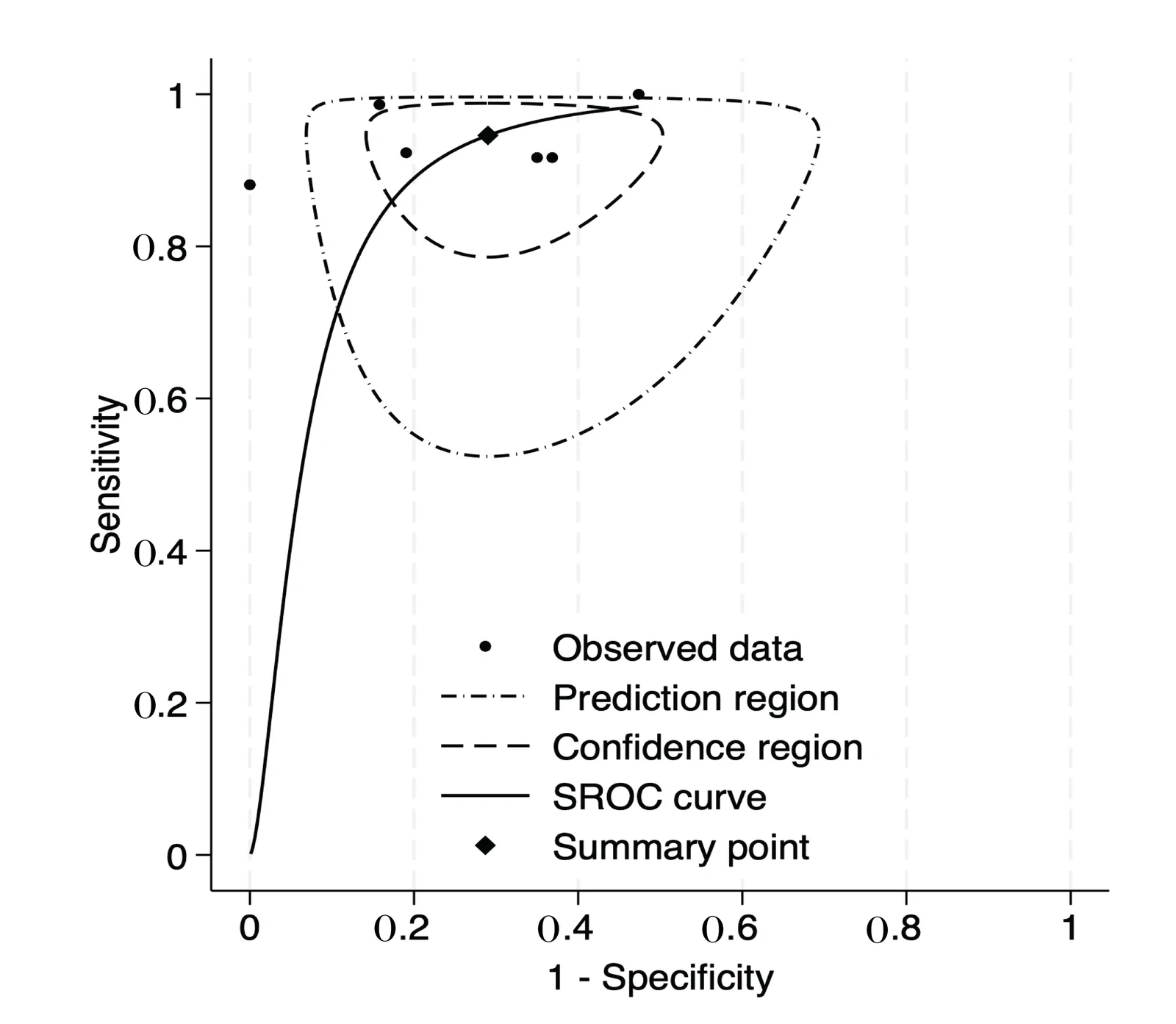
The summary receiver operating characteristic (sROC) curve illustrates the relationship between sensitivity and specificity, accounting for different cutoffs across studies. The summary point (diamond) shows average sensitivity and specificity across studies. Circles represent individual studies. The confidence region shows the confidence interval for the summary point (diamond). The prediction region indicates where the results of future studies are likely to fall.

We estimated summary measures of SE and SP as 0.95 (95% CI: 0.88, 0.98, p<0.01) and 0.71 (95% CI: 0.60, 0.80, p<0.01), respectively (Figures 2, 3). 

Heterogeneity was moderate for both SE (I2=32.68, =0.38) and SP (I2=13.17, =0.15). The following variables were studied to see if they explained the heterogeneity: study type, LUS protocol, comparison to CXR, comparison to CT scan of the chest, and age. Serial meta-regressions to determine if the (above) variable was significant and if including it reduced heterogeneity. None of them did. Upon meta-regression with study type, LUS protocol, LUS compared to CXR, LUS compared to CT scan of chest, or multivariable, we were unable to account for the observed heterogeneity; results did not substantively change (data not shown).

The summary relationship between SE and SP shown in Figure 3 accounts for different cutoffs across studies. All but two studies are within the confidence region around the summary estimate, and all but one study was within the prediction region, indicating where future studies are likely to lie. We assessed asymmetry using Deeks’ plot and tested for small study effects and publication bias using Fagan’s test. There was no evidence of bias in publication/small study effects (p=0.87) or asymmetry among these studies.

Discussion

This meta-analysis confirms the clinical applicability of LUS in diagnosing pneumonia in adult patients admitted to the ICU. Across six studies with a total of 275 patients with pneumonia, the estimated SE and SP were 0.95 and 0.71, respectively. The high SE reported across all the studies makes LUS application a reliable tool to investigate and rule out patients with suspected pneumonia in the ICU. The SP was moderate, making the use of LUS limited in ruling in pneumonia in the ICU due to relatively larger false positive results. These results are notable since no prior meta-analysis focusing on adult ICU patients exists for such a comparison. We chose to compare the accuracy of LUS to multiple modalities used in the diagnosis of pneumonia to mimic real-world applications and practice. Recently, Orso et al. [11] studied LUS and compared its accuracy to CXR only, with SE of 0.93 and SP of 0.83, and these results are close to the results of ours, despite their stricter inclusion criteria for pneumonia. Other meta-analyses done in acute non-ICU settings, such as emergency departments or general hospital wards, show results closer to our SE but greater SP. Long et al. [9] in 2017 studied over 1,500 subjects in 12 studies and found the SE for LUS to be 0.88 and the SP to be 0.86. An earlier meta-analysis by Chavez et al. [29], which included 10 studies with a sample size of 1172 participants, combining emergency department and ICU adult patients, concluded an SE of 0.94 and an SP of 0.96. 

As illustrated by all studies, including our meta-analysis, LUS demonstrates consistently superior SE, which highlights its diagnostic utility as a powerful tool in managing patients with pneumonia in the ICU. These findings support the use of LUS in acute and resource-limited settings. The rapid application of LUS can optimize supportive care and antibiotic use, which can decrease uncertainty and improve patient outcomes. The moderate SP observed in our meta-analysis may reflect the heterogeneity used as the standard for the diagnosis of pneumonia. They included a combination of clinical data, history and physical exam, laboratory data, biological markers, CXR, and CT scan of the chest. All these variables can give different results and, on many occasions, depend on the treating clinician and their final interpretation and diagnosis of pneumonia. The studies included in our meta-analysis were done in different countries and different ICU settings, which can influence LUS performance and interpretation. LUS can detect early changes and small subpleural consolidations, making the SP compared to the standard of care marginal. Many ICU patients are mostly supine in position and may be on MV. In our meta-analysis, Babu et al. [23] enrolled patients with suspicion of VAP on MV, where they used clinical exams and laboratory tests for diagnostic testing and compared this to LUS exams, with SE of 1 and SP of 0.53. Berlet et al. [24] conducted a prospective observational study that included patients on MV, and those were assigned a score depending on clinical and combined diagnostic testing, including lower respiratory tract specimens, and were divided into groups of patients who developed VAP and those with no VAP. Those results were compared with LUS performance, which showed an SE of 0.92 and a lower SP of 0.65. This was explained by the abundance of consolidations caused by conditions other than inflammation, as what happens when compression atelectasis can be caused by pleural effusion or post-obstructive atelectasis secondary to aspiration or thick secretions. Bitar et al. [25] studied patients in a single ICU setting and compared radiological reports, clinical progress, inflammatory markers, and microbiology studies to the LUS exam and reported an SE of 0.99 and an SP of 0.84. CT scans were performed in about half of their cohort, showing even more correlation with LUS compared to the CXR performed. Dhawan et al. [26] studied the accuracy of LUS in patients diagnosed with pneumonia in the ICU; almost half were on MV, and their findings showed LUS to be accurate in detecting pneumonia with an SE of 0.88 and SP of 1, and those confirmed by LUS were also evaluated by CT chest, showing a closer correlation compared to the CXR. Mancusi et al. [27] evaluated the use of LUS in the diagnosis of pneumonia and compared it to clinical exams and biological testing, as well as CXR and CT chest, and reported the accuracy of LUS with an SE of 0.92 and an SP of 0.81 with close resemblance to other observational studies. Zhou et al. [28] studied the correlation between LUS and pneumonia diagnosed using procalcitonin, respiratory cultures, and CT chest and found that LUS was accurate with SE 0.92 and SP of 0.63 when used alone, but the SP improved to 0.85 when combined with procalcitonin, where the consolidations noted were not attributed to inflammatory reasons. 

When combining studies with different practice guidelines and different protocols used in the diagnosis of pneumonia, this can lead to an increase in between-study variation and heterogeneity, and in our meta-analysis, it could have contributed to the variability and marginal SP [30]. Furthermore, these studies were conducted across multiple countries with varying practice guidelines; in addition, blinding of LUS interpreters was not reported in any included study, introducing potential performance bias. LUS may be challenging to perform in ICU patients, since most are supine, limiting accessible examination zones, as well as predisposing to rapid atelectasis, which could be difficult to distinguish from a pneumonic consolidation [31]. This is particularly true in the dorsal lung zones, potentially reducing SP. In summary, diagnostic imaging with LUS in the ICU could be challenging due to patient instability, restricted positioning, and MV, but it can also have the benefit of decreasing risks of transporting patients for other studies [32], as well as decreasing the need and cost for daily CXR [33]. 

Clinical implications and future perspectives: Overall, this meta-analysis shows that LUS is a useful tool in the diagnosis and assessment of pneumonia in the ICU. Its non-invasive nature and bedside applicability make it a valuable tool to assist clinicians in busy environments by enabling earlier and precise diagnosis and may help identify the disease process and optimize resource utilization and radiation exposure. 

Limitations

This meta-analysis has several limitations. Moderate heterogeneity was observed, with an I² of 32.6 for SE and 13.1 for SP, likely attributable to differences in study design, reference standard definitions, LUS protocols, and equipment used. Variation in ICU settings, geographic location, and operator expertise may have further contributed. Notably, heterogeneity may also reflect the real-world variability inherent in clinical practice, since the diagnosis of pneumonia, depending on the pathogen, can result in different ultrasound patterns as seen in viral versus bacterial pneumonia, leading to an increase in heterogeneity of the study. The high SE observed raises the possibility of non-blinding bias, which cannot be ruled out and may overestimate LUS performance. Variability in reference standards across studies may have introduced errors in estimating the pooled diagnostic accuracy of LUS.

## Conclusions

LUS demonstrates high SE and moderate SP for diagnosing pneumonia in adult ICU patients, supporting its role as a valuable bedside diagnostic tool. Its non-invasive nature, rapid applicability, and independence from patient transport make it particularly well-suited for critically ill patients who may be hemodynamically unstable, mechanically ventilated, or unable to be safely moved for conventional imaging. Because LUS has moderate SP, a positive finding should always be interpreted alongside the patient's clinical presentation, laboratory data, and other imaging rather than used in isolation to establish a definitive diagnosis of pneumonia. Conducting future studies, in addition to further data collection and analysis, is needed to further understand and optimize its diagnostic performance and guidelines for its use in the ICU setting to better manage patients with pneumonia.
